# *Amburana cearensis* seed extract stimulates astrocyte glutamate homeostatic mechanisms in hippocampal brain slices and protects oligodendrocytes against ischemia

**DOI:** 10.1186/s12906-023-03959-0

**Published:** 2023-05-11

**Authors:** Rafael Short Ferreira, Paulo Roberto Ribeiro, Juliana Helena Castro e Silva, Juliana Bender Hoppe, Monique Marylin Alves de Almeida, Beatriz Correia de Lima Ferreira, Gustavo Borges Andrade, Suzana Braga de Souza, Luzimar Gonzaga Ferdandez, Maria de Fátima Dias Costa, Christianne Gazzana Salbego, Andrea Domenico Rivera, Aline Longoni, Adriano Martimbianco de Assis, Francesca Pieropan, José Cláudio Fonseca Moreira, Silvia Lima Costa, Arthur Morgan Butt, Victor Diogenes Amaral da Silva

**Affiliations:** 1 Laboratory of Neurochemistry and Cell Biology, Department of Biochemistry and Biophysics, Institute of Health Sciences, Federal University of Bahia - UFBA, Salvador, Bahia 40110-902 Brazil; 2 School of Pharmacy and Biomedical Sciences, University of Portsmouth, St Michael’s Building, White Swan Road, Portsmouth, PO1 2DT UK; 3 Metabolomics Research Group, Department of Organic Chemistry, Chemistry Institute, Federal University of Bahia, Salvador, Bahia, Brazil; 4 Department of Biochemistry, Institute of Basic Health Sciences, Federal University of Rio Grande Do Sul, Porto Alegre, Brazil; 5 Biochemistry, Biotechnology and Bioproducts Laboratory, Institute of Health Sciences, Federal University of Bahia, Salvador, Bahia, Brazil; 6 Health Sciences Centre, Post-Graduate Program in Health and Behaviour, Catholic University of Pelotas, Pelotas, Brazil

**Keywords:** *Amburana cearensis*, Hippocampus, Oligodendrocyte, Astrocyte, Stroke

## Abstract

**Background:**

Stroke is a leading cause of death and disability worldwide. A major factor in brain damage following ischemia is excitotoxicity caused by elevated levels of the neurotransmitter glutamate. In the brain, glutamate homeostasis is a primary function of astrocytes. *Amburana cearensis* has long been used in folk medicine and seed extract obtained with dichloromethane (EDAC) have previously been shown to exhibit cytoprotective activity in vitro. The aim of the present study was to analyse the activity of EDAC in hippocampal brain slices.

**Methods:**

We prepared a dichloromethane extract (EDAC) from A. cearensis seeds and characterized the chemical constituents by 1H and 13C-NMR. Hippocampal slices from P6-8 or P90 Wistar rats were used for cell viability assay or glutamate uptake test. Hippocampal slices from P10-12 transgenic mice SOX10-EGFP and GFAP-EGFP and immunofluorescence for GS, GLAST and GLT1 were used to study oligodendrocytes and astrocytes.

**Results:**

Astrocytes play a critical role in glutamate homeostasis and we provide immunohistochemical evidence that in excitotoxicity EDAC increased expression of glutamate transporters and glutamine synthetase, which is essential for detoxifying glutamate. Next, we directly examined astrocytes using transgenic mice in which glial fibrillary acidic protein (GFAP) drives expression of enhanced green fluorescence protein (EGFP) and show that glutamate excitotoxicity caused a decrease in GFAP-EGFP and that EDAC protected against this loss. This was examined further in the oxygen–glucose deprivation (OGD) model of ischemia, where EDAC caused an increase in astrocytic process branching, resulting in an increase in GFAP-EGFP. Using SOX10-EGFP reporter mice, we show that the acute response of oligodendrocytes to OGD in hippocampal slices is a marked loss of their processes and EDAC protected oligodendrocytes against this damage.

**Conclusion:**

This study provides evidence that EDAC is cytoprotective against ischemia and glutamate excitotoxicity by modulating astrocyte responses and stimulating their glutamate homeostatic mechanisms.

**Supplementary Information:**

The online version contains supplementary material available at 10.1186/s12906-023-03959-0.

## Background

The plant *Amburana cearensis* (Allemao) A.C. Sm. is used traditionally as tea, decoct and syrup for the treatment of various ailments, including headache, migraine and inflammation [1–3]. Previous work by our group has demonstrated neuroprotective effects against glutamate-mediated excitotoxicity in PC12 cells and primary cultures of cerebellar cells of the dichloromethane extract of *A. cearensis* seeds (EDAC) containing coumarin, ethyl ester, methyl esters and γ -sitosterol [4, 5]. Additionally, Amburoside A, a glucoside from *A. cearensis* trunk bark, has been shown to exhibit antioxidative and neuroprotective effects against 6-OHDA toxicity *in* rat mesencephalic cell cultures [6]. Together, these studies support the cytoprotective potential of compounds from *A.* *cearensis* in the CNS, but their effects on glial cells in brain tissues were unresolved.

Astrocytes and oligodendrocytes are the main glial cell types in the CNS. Oligodendrocytes are specialised to myelinate axons and are essential for the rapid conduction of neural impulses. Notably, oligodendrocytes are one of the most affected brain cells in ischemia, which results in devastating effects on CNS function [7–10]. For example, oligodendrocytes in the hippocampus are damaged following transient focal cerebral ischemia [11], and have been shown to be highly susceptible to oxygen–glucose deprivation (OGD), a model of ischemia-hypoxia that involves glutamate excitotoxicity [12–14]. Glutamate homeostasis is a primary function of astrocytes and they have been shown to protect oligodendrocytes following hypoxia and reperfusion-induced damage by uptake of excess glutamate [15, 16]. Astrocytes respond to glutamate excitotoxicity in a complex spatio-temporal manner that is highly context specific. Astrocyte pathological changes may be marked by a loss of processes and possibly cell death in acute phases and close to the lesion site [17]. At the other extreme, astrocytes may become hypertrophic, with increased glial fibrillary acidic protein (GFAP) and process branching [18, 19]. Furthermore, depending on the context, there may be either up- or down-regulation of astrocyte glutamate homeostasis, specifically altered expression of glutamine synthetase (GS), glutamate transporter 1 (GLT1) and glutamate aspartate transporter (GLAST) [18, 19]. Hence, modification of astrocyte responses to glutamate excitotoxicity has considerable therapeutic potential for protecting the brain in multiple pathologies and there is increasing interest in natural products in this context [20, 21].

In this study, we examined the cytoprotective potential of *A. cearensis* seed extracts (EDAC) ex vivo in hippocampal brain slices. We show it is cytoprotective for astrocytes and oligodendrocytes against ischemic and glutamate mediated excitotoxicity by maintaining astrocyte glutamate homeostatic mechanisms.

## Materials and methods

### Animals and *t*issues

Mice and rats were used in this study. Mice were used in the University of Portsmouth (UK) approved by the University of Portsmouth Animal Welfare and Ethical Review Board (P93781054) in compliance with the revised Animals (Scientific Procedures) Act 1986. Rats were used in the University of Bahia (Brazil) approved by the Ethics Committee on the Use of Animals (CEP-UFRGS, protocol number 20005) and in compliance with the “Principles of Care for Laboratory Animals”, publication number 85–23, of the National Institute of Health (NIH). The study is reported in accordance with ARRIVE guidelines (https://arriveguidelines.org). Mice were transgenic reporter strains in which the expression of Enhanced Green Fluorescent Protein (EGFP) is driven by the astrocyte gene Glial Fibrillary Acidic Protein (GFAP) [22] or the oligodendroglial gene SOX10 [8]. Rats were of the Wistar strain. All animals were euthanised by carbon dioxide (mice) or decapitation (rats) and brains removed; for acute experiments, brains were placed immediately in ice chilled artificial cerebrospinal fluid (aCSF, containing in mM 133 NaCl, 3 KCl, 1.5 CaCl_2_, 1.2 NaH_2_PO_4_, 1.0 MgCl_2_, 10 D-glucose, 10 HEPES, pH 7.3), and for organotypic culture brains were placed in chilled balanced Hank's solution (HBSS, Invitrogen, containing in mM 137 NaCl, 0.6 Na_2_HPO_4_, 3.0 NaHCO_3_, 5.0 KCl, 0.4 KH_2_PO_4_, 1.26 CaCl_2_, 0.9 MgSO_4_, and 5.55 glucose, 20 HEPES, pH 7.2).

### Preparation of extracts of *A. cearensis* seeds (EDAC)

Seeds of *Amburana cearensis* were purchased from a general store in the city of Feira de Santana, Bahia, Brazil, and checked for authenticity by comparison with seeds deposited in the Herbarium of the Biology Institute of the Federal University of Bahia, with the number 13734. All methods with *A. cearensis* seeds were carried out in accordance with the guidelines and standards of the University of Bahia, under registration in the National System for the Management of Genetic Heritage and Associated Traditional Knowledge (SisGen) under the number A73B242. *A. cearensis* seed extracts (EDAC) were prepared as described previously [5]. In brief, *A. cearensis* seeds were slow dried at 40° C for 24 h and the EDAC extract was prepared by subjecting dried and fine-ground seeds to maceration in dichloromethane for 72 h, after which the solvent was completely removed in a rotary evaporator. Then, the crude extract was dissolved in a mixture of ethanol and water (8:2) and this solution partitioned three times with dichloromethane (DCM) to obtain EDAC precipitate, which was recrystallized in ethanol and the main component identified as coumarin by nuclear magnetic resonance (Supplementary Fig. 1 A-F and Supplementary Table 1); the pure coumarin was used as the standard for quantification of extracts. Two to five milligrams of coumarin and the EDAC extract were dissolved in Deuterated Chloroform (CDCl_3_) (500 μL), filtered, and transferred to 5-mm nuclear magnetic resonance (NMR) tubes. ^1^H and ^13^C-NMR spectra were acquired at 20 °C using Bruker AVANCE III spectrometer (600 MHz for ^1^H-NMR and 125 MHz for ^13^C-NMR). Tetramethylsilane (TMS) was used as internal standard. NMR spectra were processed at MestreNova [23].

For quantification purposes, we used the noesypr1d (Bruker standard) pulse sequence, performed with 64 scans (ns) with 128 k points during acquisition (td) in a spectral window (sw) of 15 ppm and with a receiver gain (rg) of 203, 1.0 s between each acquisition (d1) and this had a total time (aq) of approximately 7.27 s. The power attenuation for the pre-saturation (PLdB9) was 52.27 dB. Total acquisition time of each spectrum was 09 min and 31 s. Spectra were processed with 128 k (SI) using an exponential multiplication with a lb of 0.3 Hz and phase and baseline automatic correction. NMR-based quantification of coumarin was performed based on the integration of two doublets at 6.43 (d, 1H, 9.56 Hz) and 7.72 (d, 1H, 9.48 Hz) attributed to C2 and C3, respectively [24].

For biological experiments EDAC was diluted in dimethyl sulfoxide (DMSO) to make a 50 mg/mL stock solution that was stored at 4 °C protected from light and final concentrations of EDAC (0.1, 1 or 10 µg/mL) were prepared fresh from stock solution prior to each experiment, with vehicle (DMSO) being used as control.

### Hippocampal slices

Hippocampal slices were prepared as detailed previously [25] with some modifications. In brief, the hippocampus in each hemisphere was isolated from the brain and transverse slices of hippocampus with a thickness of 400 µm were acquired using a McILwain Tissue Chopper. For the oxygen and glucose deprivation (OGD) experiments, slices were placed directly into aCSF at 37 °C, and for organotypic culture, slices were placed immediately into cold HBSS.

### High glutamate treatment of organotypic hippocampal slice cultures

Hippocampal slices were placed on membrane inserts (Millicell®-CM 0.4 μm, Millipore) and placed in wells containing culture medium comprising 50% minimum essential medium (MEM, Invitrogen), 25% HBSS and 25% horse serum (HS), supplemented with 36 mM glucose, 25 mM HEPES, 4 mM NaHCO_3_, 100 IU/ml penicillin G, 100 mg/ml streptomycin and 1% non-essential amino acids (Gibco™ 11,140,050), pH 7.3. Slices were maintained for two weeks at 37 °C in 5% CO_2_, with a change of medium every 3 days. On day 13, slices were treated for 24 h with medium containing elevated glutamate (1 or 3 mM) with and without EDAC (0.1, 1 or 10 µg/mL); agents were added directly to the culture medium. At the end of the experiment, slices were treated for propidium iodide (PI) labelling or placed in fixative prior to immunolabelling.

### Oxygen–glucose deprivation (OGD) of acute hippocampal slices

Hippocampal slices were placed for 1 h in *a*CSF containing vehicle (DMSO) or EDAC (1 or 10 µg/mL) and then incubated in the same solution for 1 h in normal oxygen and glucose (OGN) or in oxygen–glucose deprivation (OGD), as described previously [8]. For OGN, slices were maintained in aCSF containing 10 mM D-glucose in a 95% O_2_/5% CO_2_ atmosphere, and for OGD slices were maintained in aCSF with 10 mM sucrose in a 95% N_2_/5% CO_2_ atmosphere; all experiments were performed at 37 °C and pH was maintained constant at pH 7.3 using 10 mM HEPES buffer. At the end of the experiment, slices were placed in fixative prior to confocal microscopy.

### Propidium iodide (PI) labelling

PI labelling was used as measure of cell death as previously described [21], following treatment of organotypic slices with glutamate and/or EDAC, by incubation for 30 min with 5 μg/mL PI in aCSF at 37ºC and in a humidified atmosphere of 5%CO_2_. After incubation, slices were imaged using a Nikon Eclipse TE 300 Inverted fluorescence microscope (Nikon Instruments Inc., Americas) with a × 4 objective and CCD camera (Visitron Systems, Puchheim, Germany). Images were analysed using Scion Image software (Scion Corporation; www.scioncorp.com), using the "density slice" function to measure the area of PI fluorescence, as a percentage of the total area of each slice.

### Glutamate uptake in hippocampal brain slices

Uptake of radiolabeled glutamate was measured as previously described [21]. Hippocampal slices (*n* = 6) from rats aged 90 days old were first incubated for 1 h at 37 °C in HBSS containing EDAC (1 μg/mL or 10 μg/mL) or DMSO vehicle in controls, followed by 1 h in HBBS containing elevated glutamate (3 mM) with or without EDAC (1 or 10 μg/mL). After the treatments, slices were washed with buffer and incubated at 37 °C in HBSS containing 0.33 μCi [^3^H]L-glutamate (PerkinElmer, Boston, MA, USA). After 7 min, the experiment was stopped with two ice-cold washes of HBSS, then 0.5N NaOH was immediately added and the slices were kept overnight. Radioactivity was measured using a liquid scintillation counter (Hidex 300 SL, Mikrotek Laborsysteme, Overath, Germany). Na^+^-independent glutamate uptake was measured using the same protocol except that NaCl in the medium was replaced with N-methyl-D-glucamine throughout treatments and the experiment was performed at 4 °C. Na^+^-dependent uptake was calculated as the difference between total and Na^+^-independent [^3^H]L-glutamate uptake and results were expressed as a percentage of the control.

### Immunolabelling

Hippocampal slices were washed 3 times with 0.01 M phosphate buffer saline (PBS) and fixed in 4% paraformaldehyde (PFA) for 1 h at room temperature (RT). At the end of fixation, the slices were washed 3 times with PBS and then stored at 4 °C in PBS containing 0.05% sodium azide until use. Prior to immunolabelling, slices were washed 3 times with PBS for 10 min, then incubated overnight with 1% Triton X-100 in PBS at 4 °C, followed by a blocking stage using 20% bovine serum albumin (BSA) diluted in PBS (0.01 M) and containing 0.1% Triton (PBS-T) for 3 h at RT. Slices were then incubated overnight with primary antibody diluted in PBS-T and 1% normal goat serum (NGS): rabbit anti-NeuN antibody (1: 200, Cell Signalling Technology, D4G4O # 24,307), guinea pig anti-GLT1 antibody (1:5000, Merck Millipore Chemicon International, ab1783), rabbit anti-GLAST antibody (1: 150, Abcam, ab416), or rabbit anti-GS (1:5000, Abcam, ab49873). Following washes in PBS-T, slices were incubated in appropriate secondary antibodies for 3 h at RT, namely goat anti-guinea pig Alexa-fluor 568 or goat anti-rabbit Alexa-Fluor 647 (both at 1:500, Invitrogen), in addition to the nuclear chromatin dye Hoechst 33,342 (1:500, Fisher, 11,544,876). Lastly, slices were washed in PBS-T (0.1%) and mounted in Fluoromount G (Invitrogen—REF 00–4958-02).

### Image acquisition and analysis

Images were acquired using a Zeiss LSM 710 confocal microscope or LEICA SP5 AOBS confocal microscopy at 1 µm z-stack intervals and all acquisition parameters were maintained constant between experiments. Images were obtained from edge to edge of the hippocampal CA1 region; all slices were imaged and all images were included in analyses. The relative fluorescence intensities of GFAP-EGFP and immunostaining for GLT1, GLAST and GS were measured in images captured using a × 20 objective and from edge to edge of the CA1 region using ImageJ-Win64. Astrocyte morphology was analysed using the method described previously [26], in which cells were imaged using a × 40 oil immersion objective and z-stacks with 1 µm intervals, and binary reconstructions were made using ImageJ-Win64 with the aid of the Analyze Skeleton plugin (2D/3D); cells were analysed for the number of process terminals and branch lengths, to provide a measure of morphological complexity. Analysis of oligodendrocyte cell density was determined in images captured using a × 20 objective and by counting the number SOX10-EGFP^+^ cells in a constant FOV (100 × 200 µm) using ImageJ-Win64; process and non-process bearing SOX10-EGFP + cells were distinguished and data was expressed as the total number and % of process bearing SOX10-EGFP + cells.

### Statistical analysis

A minimum of 3 independent experiments were performed for each analysis, each with a minimum of 3 slices (exact ‘n’ values given in each experiment). The results were analysed using GraphPad Prism 8.3.0 (California, USA) and recorded as mean or median ± SEM (standard error of the mean). Statistical significance was determined by one-way analysis of variance (ANOVA), followed by the Tukey post-hoc test for parametric data, and Kruskal-Walis test with Dunns Multiple Comparison or Mann–Whitney post hoc tests for non-parametric data. Values of *p* < 0.05 were considered statistically significant.

## Results

### Coumarin is the main component of EDAC

Coumarin was identified as the main constituent of the EDAC extract by ^1^H and ^13^C NMR (Supplementary Fig. 1 A-F) [27]. This compound showed two doublets at 6.43 (d, 1H, 9.56 Hz) and 7.72 (d, 1H, 9.48 Hz) attributed to C2 and C3, respectively. It also showed two double doublets at 7.49 (dd, 1H, 1.48 e 7.72) and 7.30 (dd, 1H, 1.12 e 7.64 Hz) attributed to C5 and C6, respectively. In addition, it showed one triple doublet at 7.54 (td, 1H, 1.2 e 8.68 Hz) attributed to C7 and a double triplet at 7.34 (dt, 1H, 0.44 e 8.36 Hz) attributed to C8. ^1^H and ^13^C NMR signals were consistent with the literature [27] and ^13^C NMR spectrum of coumarin [28] and allowed us to undoubtedly assign the main compound of EDAC extract as coumarin (Supplementary Table 1). NMR-based quantification showed that EDAC extract contain 374.46 ± 85.05 mg/g of coumarin.

### EDAC protects against glutamatergic excitotoxicity and stimulates glutamate uptake in hippocampal slices

**Fig. 1:**
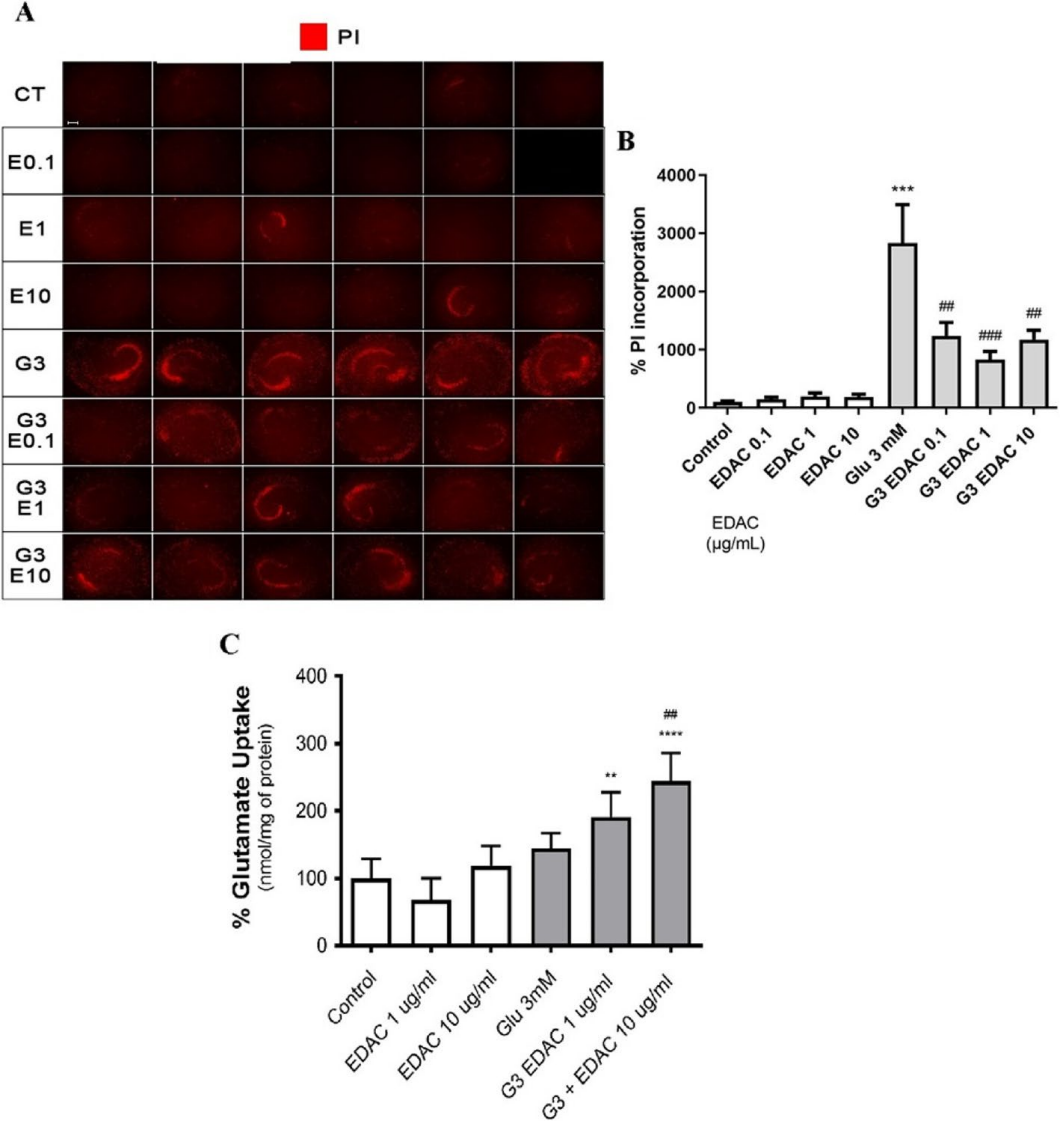
EDAC is cytoprotective against raised glutamate and stimulates glutamate uptake. Hippocampal slices from Wistar rats were treated with different concentrations of EDAC (as indicated) or DMSO vehicle in controls, for 1 h, followed by 3 mM glutamate (G3) with or without EDAC. **A**,** B** Hippocampal slices were stained with propidium iodide (PI), a marker for dying cells; images from *n* = 6 slices per experimental group (**A**) were analysed for PI relative fluorescence as a percentage of the total area of each slice and data plotted as mean ± SEM. **C** Glutamate uptake was measured following the different treatments by incubating slices with radiolabelled glutamate for 7 min and measuring the amount taken up into tissue (nmol/mg of protein); data are expressed as a % of control and plotted as mean ± SEM (*n* = 12 per experimental group). Data were tested for significance using one-way ANOVA, followed by Tukey’s post-hoc tests; in B and C, **p < 0.01 and ***p < 0.0001 indicate comparisons with control, and ##*p* < 0.01 and ###*p* < 0.001 indicate comparisons with 3 mM glutamate

The effect of EDAC on glutamate-mediated excitotoxicity and glutamate uptake were measured in hippocampal slices from postnatal rats aged P6-8. Hippocampal slices were maintained in organotypic culture for 13 days in vitro (DIV) and then treated for 24 h with 3 mM glutamate plus DMSO vehicle or EDAC at 0.1, 1 or 10 µg/mL (approximately equivalent to 0.0375, 0.375 and 3.75 µg/ml coumarin); controls were not treated with glutamate (Fig. 1). PI staining was used as a measure of cell death and little or no PI labelling was observed in untreated controls or following treatment with EDAC, indicating the viability of the hippocampus slice cultures and that EDAC is not cytotoxic at any of the concentrations used (Fig. 1A, B). In contrast, treatment with 3 mM glutamate markedly increased PI staining compared to untreated controls (Fig. 1A) and this was confirmed by quantification, with a significant (*p* < 0.0001) 30-fold increase in PI relative fluorescence intensity compared to controls (Fig. 1B). The glutamate-induced increase in PI staining was significantly reduced by more than half in EDAC treatment (Fig. 1A), for example from 2834 ± 1865% in 3 mM glutamate to 832 ± 367% in 3 mM glutamate plus 1.0 µg/mL EDAC (Fig. 1B, individual *p* values shown on the graph). The results demonstrate that EDAC protects against glutamate-mediated excitotoxicity.

Extracellular glutamate levels are tightly regulated in the brain via Na^+^-dependent transporters and so we next measured the effects of EDAC on the uptake of radiolabeled glutamate in acute hippocampal slices. In each of the different treatment groups, the amount of ^3^H-L-glutamate in the tissue was determined after 2 h in slices maintained in the presence or absence of sodium, to provide a measurement of Na^+^-dependent uptake expressed as a % of the control background tissue level in untreated slices (Fig. 1C). Treatment with EDAC (1 or 10 μg/ mL) or 3 mM glutamate had no significant effect on glutamate uptake compared to controls. In comparison, EDAC significantly increased glutamate uptake in slices treated with 3 mM glutamate, from 144.2 ± 22.63% in 3 mM glutamate, to 191.3 ± 36.05 in 1 μg/mL EDAC + glutamate and 244.2 ± 41.53% in 10 μg/mL EDAC + glutamate (Fig. 1C, individual *p* values shown on the graph). The results indicate that stimulation of glutamate uptake is a key mechanism for the cytoprotective action of EDAC against glutamate excitotoxicity.

### EDAC increases expression of astrocyte glutamate homeostatic mechanisms in glutamatergic excitotoxicity

**Fig. 2:**
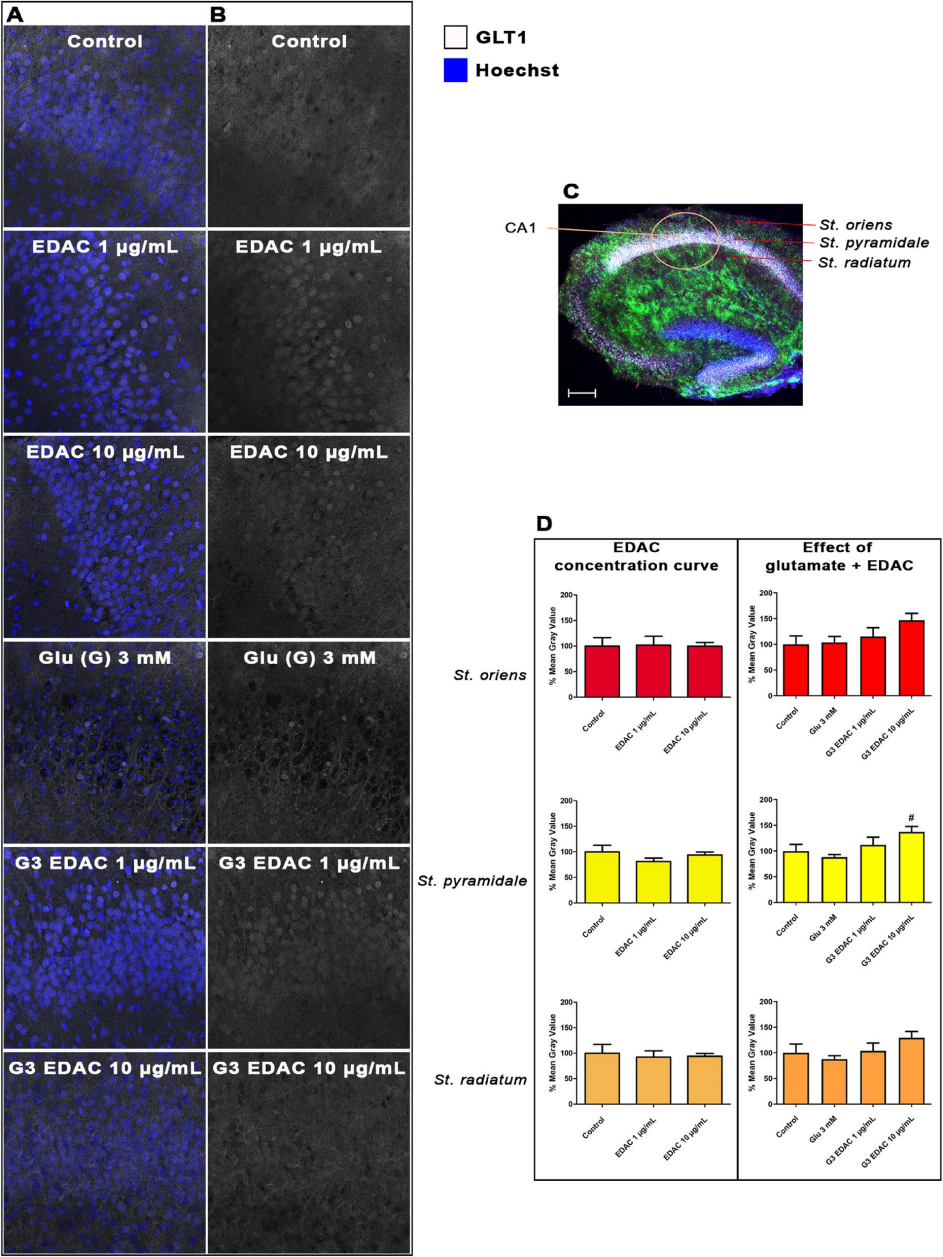
Effects of EDAC on glutamate transporter GLT1 expression in hippocampal organotypic cultures. Hippocampal slices from P10-12 mice were maintained in organotypic culture for 13 DIV, then treated for 24 h with 3 mM glutamate with or without EDAC (1 or 10 μg/mL) and then nuclei were stained with Hoechst 33,342 (**A**) and immunolabeled for GLT1 (**B**), controls did not receive glutamate. **A** Representative confocal images from the different experimental groups, as indicated, immunofluorescence labelled for GLT1 (white) and counterstained with Hoechst for cell nuclei (blue). **C** Representative confocal images from the different experimental groups, as indicated, immunofluorescence labelled for GLT1 (white). Scale bar 20 μm. **C** Confocal image of hippocampal slice (taken using × 5 objective) from the P6 GFAP-EGFP mouse shows the CA1 region and its layers labelled, s*trata oriens, pyramidale* and *radiatum* analyzed, which the IF intensity was analyzed. In green, GFAP positive cells, in white NeuN to label neurons, and in blue nuclei stained with Hoechst.The Scale bar 200 µm. **D** Relative fluorescence intensity of GLT1 immunostaining in the different layers of the CA1, as indicated, expressed as a % of control (DMSO), and plotted as mean ± SEM (*n* = 6 per experimental group). Data were tested for significance using one-way ANOVA, followed by Tukey’s post-hoc tests, # *p* < 0.05, represents a statistical difference compared to 3 mM glutamate

**Fig. 3:**
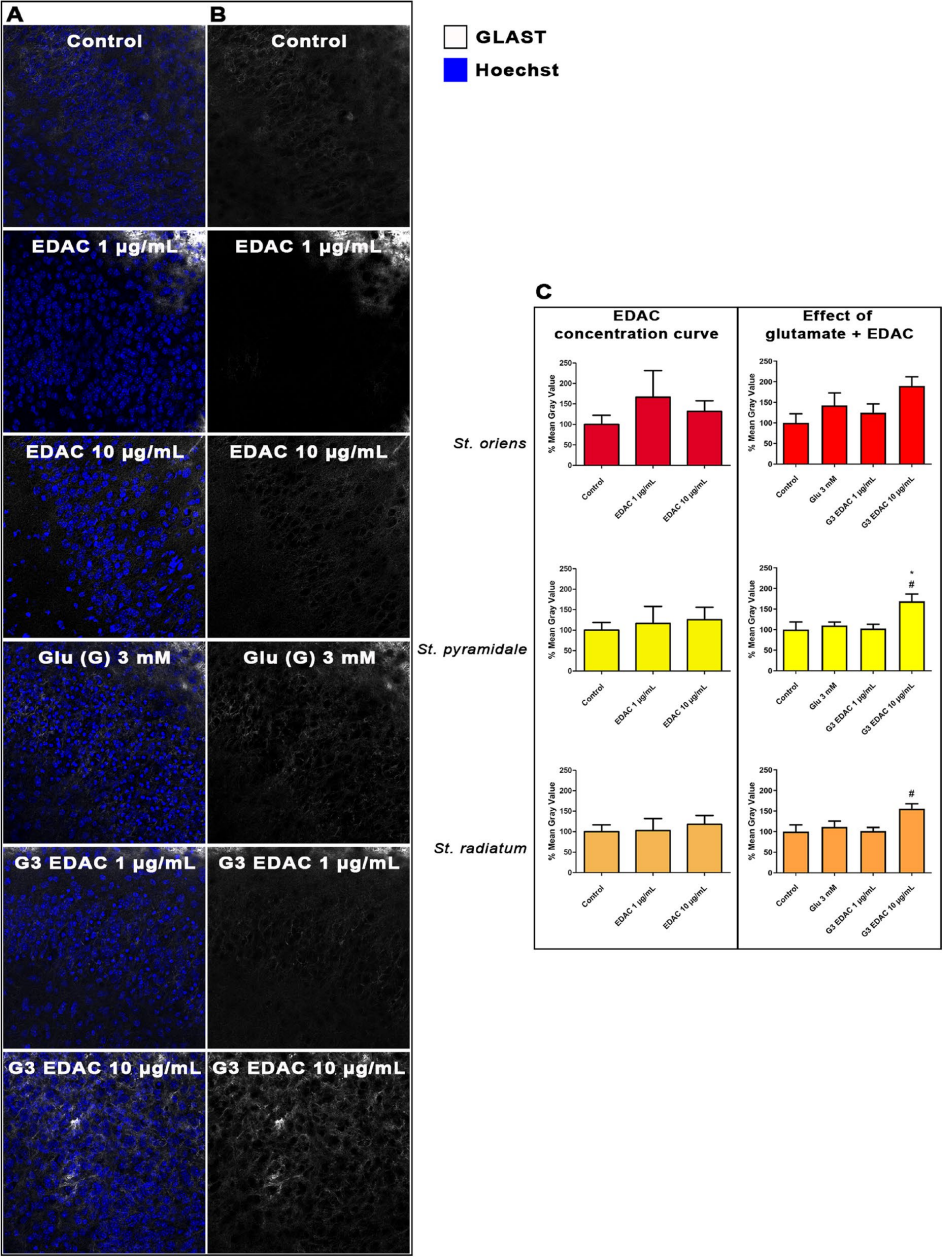
Effects of EDAC on glutamate transporter GLAST expression in hippocampal organotypic cultures. Hippocampal slices from P10-12 mice were maintained in organotypic culture for 13 DIV, then treated for 24 h with 3 mM glutamate with or without EDAC (1 or 10 μg/mL) and then immunolabeled for GLAST, controls did not receive glutamate. **A** Representative confocal images from the different experimental groups, as indicated, immunofluorescence labeled for GLAST (white) and counterstained with Hoechst for cell nuclei (blue). **B** Representative confocal images from the different experimental groups, as indicated, immunofluorescence labelled for GLAST (white). Scale bar 20 μm. **C** Relative fluorescence intensity of GLAST immunostaining in the different layers of the CA1 (s*trata oriens, pyramidale* and *radiatum*), as indicated, expressed as a % of control (DMSO), and plotted as mean ± SEM (*n* = 6 per experimental group). Data were tested for significance using one-way ANOVA, followed by Tukey’s post-hoc tests, # *p* < 0.05, represents a statistical difference compared to 3 mM glutamate

**Fig. 4:**
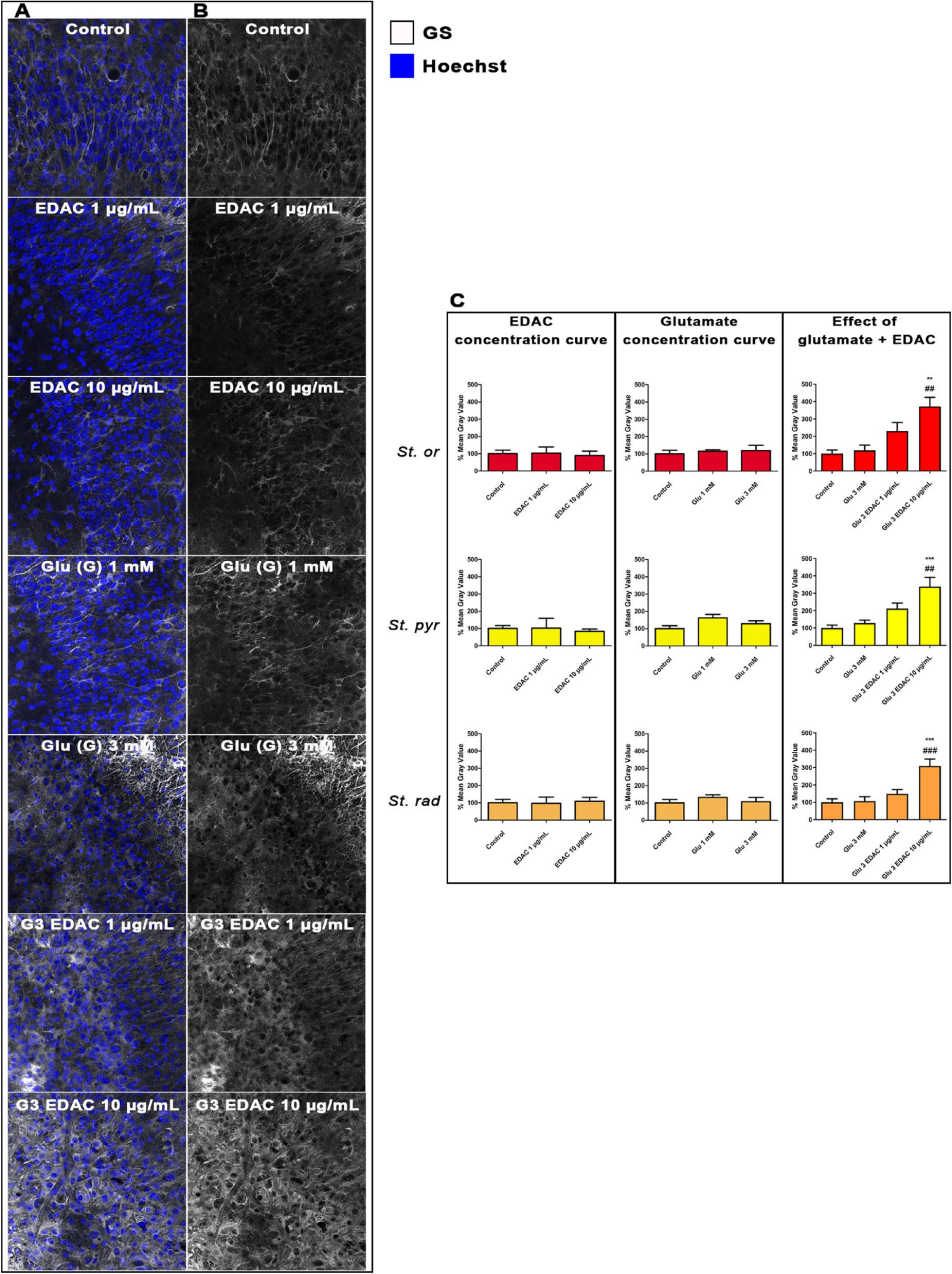
Effects of EDAC on glutamine synthetase (GS) expression in hippocampal organotypic cultures. Hippocampal slices from P10-12 mice were maintained in organotypic culture for 13 DIV, then treated for 24 h with 3 mM glutamate with or without EDAC (1 or 10 μg/mL) and then immunolabeled for GS, controls did not receive glutamate. **A** Representative confocal images from the different experimental groups, as indicated, immunofluorescence labelled for GS (white) and counterstained with Hoechst for cell nuclei (blue). Scale bar 20 μm. **B** Representative confocal images from the different experimental groups, as indicated, immunofluorescence labelled for GS (white). **C** Relative fluorescence intensity of GS immunostaining in the different layers of the CA1 (s*trata oriens, pyramidale* and *radiatum*), as indicated, expressed as a % of control (DMSO), and plotted as mean ± SEM (*n* = 5 per experimental group). Data were tested for significance using one-way ANOVA, followed by Tukey’s post-hoc tests, **p < 0.01 and ***p < 0.0001 indicate comparisons with control, and ##*p* < 0.01 and ###*p* < 0.001 indicate comparisons with 3 mM glutamate

Astrocytes are responsible for preventing glutamate accumulation in the brain and protecting against excitotoxicity by uptake primarily through glutamate transporter-1 (GLT-1), together with glutamate-aspartate transporter (GLAST), and by its conversion into non-toxic glutamine via the enzyme glutamine synthetase (GS). We therefore examined the effects of EDAC on the relative levels of immunostaining for GLT-1, GLAST and GS in hippocampal brain slices exposed to elevated (3 mM) glutamate for 24 h (Figs. 2–4). The immunostaining for both GLT-1 (Fig. 2A, B), GLAST (Fig. 3A, B), and GS (Fig. 4A, B) was observed throughout the *strata oriens, pyramidale* and *radiatum* of CA1 region (Fig. 2 C). The pattern of GLT-1, GLAST and GS expression were unaltered by treatment with 3 mM glutamate or EDAC (Figs. 2A and B, 3A and B, 4A, B), which was confirmed by quantitative analysis of relative fluorescent intensity (Figs. 2D, 3C, 4C). In comparison, treatment with 10 μg/mL EDAC in high glutamate, but not 1 μg/mL EDAC, increased immunostaining for GLT-1, GLAST and GS (Figs. 2D, 3C, 4C) and quantitative analysis revealed significant regional differences in the layers of the CA1 (Figs. 2D, 3C, 4C). In high glutamate, 10 μg/mL EDAC increased GLT1 significantly in the s*tratum pyramidale* (Fig. 2D, *p* values as indicated), and for GLAST in both the *stratum pyramidale* and *radiatum* (Fig. 3C, *p* values as indicated). The most marked changes were in GS immunostaining, which was significantly increased threefold by 10 µg/ mL EDAC in high glutamate in all layers of the CA1 (Fig. 4 C, *p* values as indicated). The results indicate that EDAC increases astrocyte glutamate homeostatic mechanisms in response to excitotoxic levels of glutamate.

### EDAC is cytoprotective for astrocytes in glutamatergic excitotoxicity and OGD

**Fig. 5:**
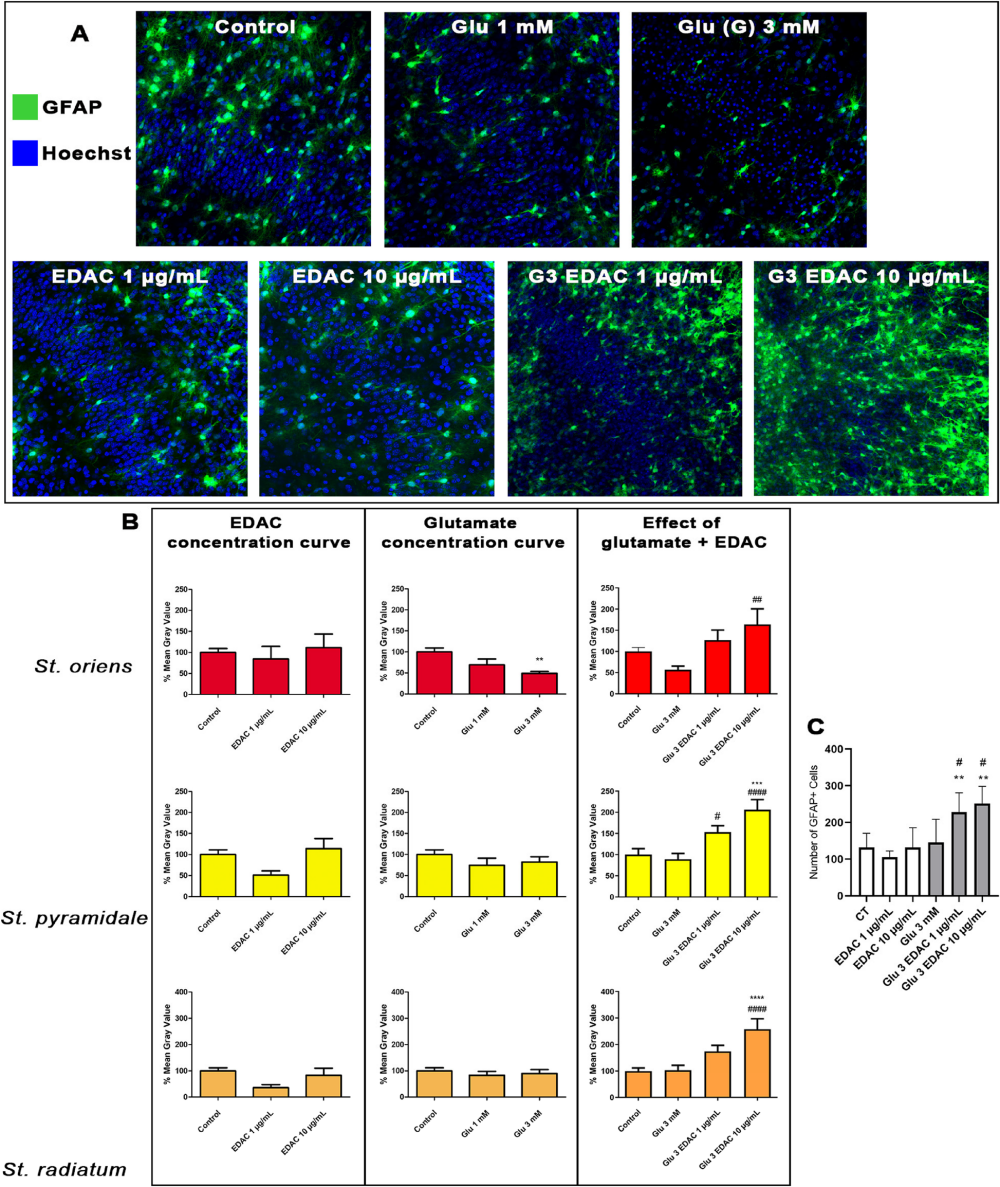
Effects of EDAC on astrocytes in raised glutamate in hippocampal slices. Hippocampal slices from P10-12 GFAP-EGFP mice were treated for 24 h with high glutamate and different concentrations of EDAC, or DMSO vehicle in controls. **A** Representative confocal images of the different treatment groups (as indicated) illustrating changes in GFAP-EGFP + astrocytes (green); cell nuclei are labelled with Hoechst (blue). Scale bar 50 µm. **B** GFAP-EGFP relative fluorescence intensity was measured in constant FOV in the CA1 s*trata oriens*, *pyramidale* and *radiatum*. Results were expressed as a % of the control and plotted as mean ± SEM (*n* = 4 for each treatment group). **C** GFAP-EGFP positive cells counting. Counts were performed of total SOX10-EGFP + cells in both s*trata oriens*, *pyramidale* and *radiatum.* Data were tested for significance using one-way ANOVA, followed by Tukey’s post-hoc tests; **p < 0.01 and ***p < 0.0001 indicate comparisons with control, and #*p* < 0.05, ##*p* < 0.01 and ###*p* < 0.001 indicate comparisons with 3 mM glutamate

**Fig. 6:**
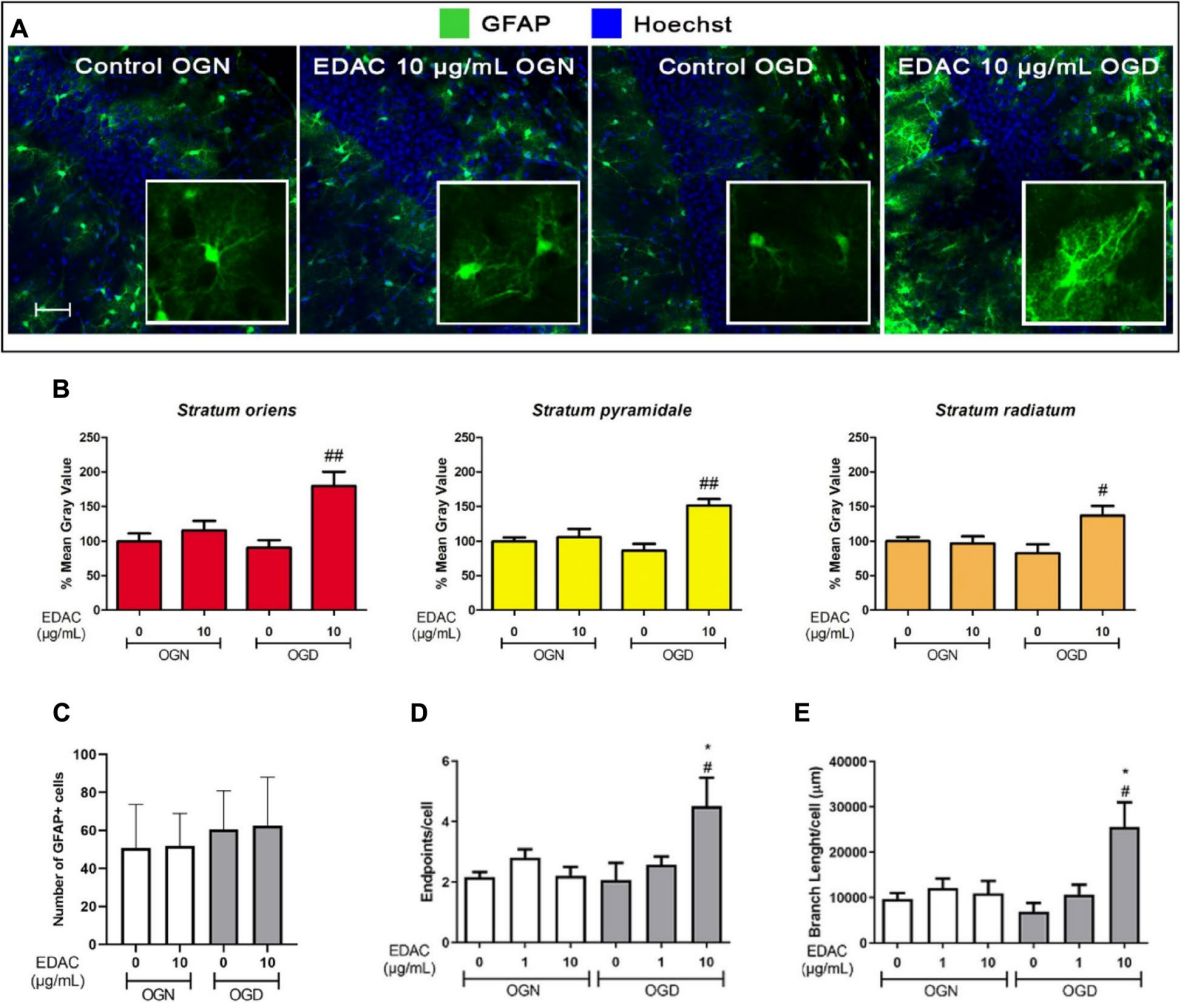
EDAC is cytoprotective for astrocytes in OGD in hippocampal slices. Hippocampal slices from P10-12 GFAP-EGFP mice were incubated in aCSF for 1 h in OGN (normal oxygen + glucose) or OGD (no oxygen or glucose), with different concentrations of EDAC, or DMSO vehicle in controls (as indicated). **A** Representative confocal images of the CA1 region of the hippocampus illustrating the effects of OGD and EDAC (10 mg/mL) on GFAP-EGFP + astrocytes (green), which were used for analysis of overall GFAP-EGFP relative fluorescence intensity (**B**); cell nuclei are labelled with Hoechst (blue). Insets are high magnification z-stacks illustrating astrocyte morphology, which were analysed for process branching (**C**). Scale bar 50 µm in main panels and 15 μm in insets. **B** GFAP-EGFP relative fluorescence intensity was measured in constant FOV in the CA1 strata (as indicated), expressed as a % of the control and plotted as mean ± SEM (*n* = 4 for each treatment group). **C** GFAP-EGFP positive cells counting. Counts were performed of total SOX10-EGFP + cells in both s*trata oriens*, *pyramidale* and *radiatum.* **D** Astrocyte morphological analysis of the number of process endpoints; (**E**) and branch lengths, plotted as mean ± SEM (*n* = 5 per treatment group). Data were tested for significance using one-way ANOVA, followed by Tukey’s post-hoc tests; *p < 0.05 indicates comparisons with OGN control, and #*p* < 0.05, ##*p* < 0.01 indicate comparisons with OGD control

The results above indicate astrocytes are altered by EDAC treatment in the face of excitotoxic levels of extracellular glutamate. Astrocytes often respond to brain damage by what is termed reactivity [29], which is often assessed by increased expression of GFAP [30]. However, studies using GFAP-EGFP reporter mice indicate astrocytes respond acutely to ischemia by a loss of cellular processes and possible cell death [17]. We therefore examined the effects of EDAC on astrocytes in hippocampal slices from GFAP-EGFP mice in response to high glutamate (Fig. 5) and OGD (Fig. 6). In organotypic cultures, there was a clear decrease in the overall expression of GFAP-EGFP after 24 h exposure to 3 mM glutamate and this was confirmed by quantification of relative fluorescence, which was significantly decreased by half in the *stratum oriens* (Fig. 4A, B; *p* values as indicated). Treatment with EDAC had no apparent effect on astrocytes in control medium, but in elevated glutamate EDAC induced a marked increase in GFAP-EGFP, more than doubling relative fluorescence in all layers of the CA1 (Fig. 5A, B; *p* values as indicated), which was associated with an increase in the number of GFAP-EGFP positive cells (Fig. 5 C).

These acute changes in astrocytes were examined further in OGD, which has been shown previously to cause astrocyte morphological atrophy and cell loss [17]. In OGN, astrocytes were distributed throughout the hippocampus and were unaltered by EDAC (Fig. 6A). In contrast, in OGD there was a clear decrease in GFAP-EGFP and treatment with EDAC in OGD resulted in a marked increase in GFAP-EGFP (Fig. 6A), which was confirmed by quantification of GFAP-EGFP relative fluorescence (Fig. 6B). There was no evident loss of GFAP-EGFP + astrocytes in OGD (Fig. 6C), and higher magnification indicated the main effect of OGD was an apparent decrease in cellular process complexity, whereas in OGD conditions EDAC clearly increased the morphological complexity of individual astrocytes (Fig. 6A, insets). This was confirmed by analysis of the number of process end-points and branch lengths, which were more than doubled by 10 µg/ mL EDAC in OGD (Fig. 6D, E; *p* values as indicated). The results indicate that EDAC is protective for astrocytes against ischemic damage.

### EDAC is cytoprotective for oligodendrocytes in OGD

**Fig. 7:**
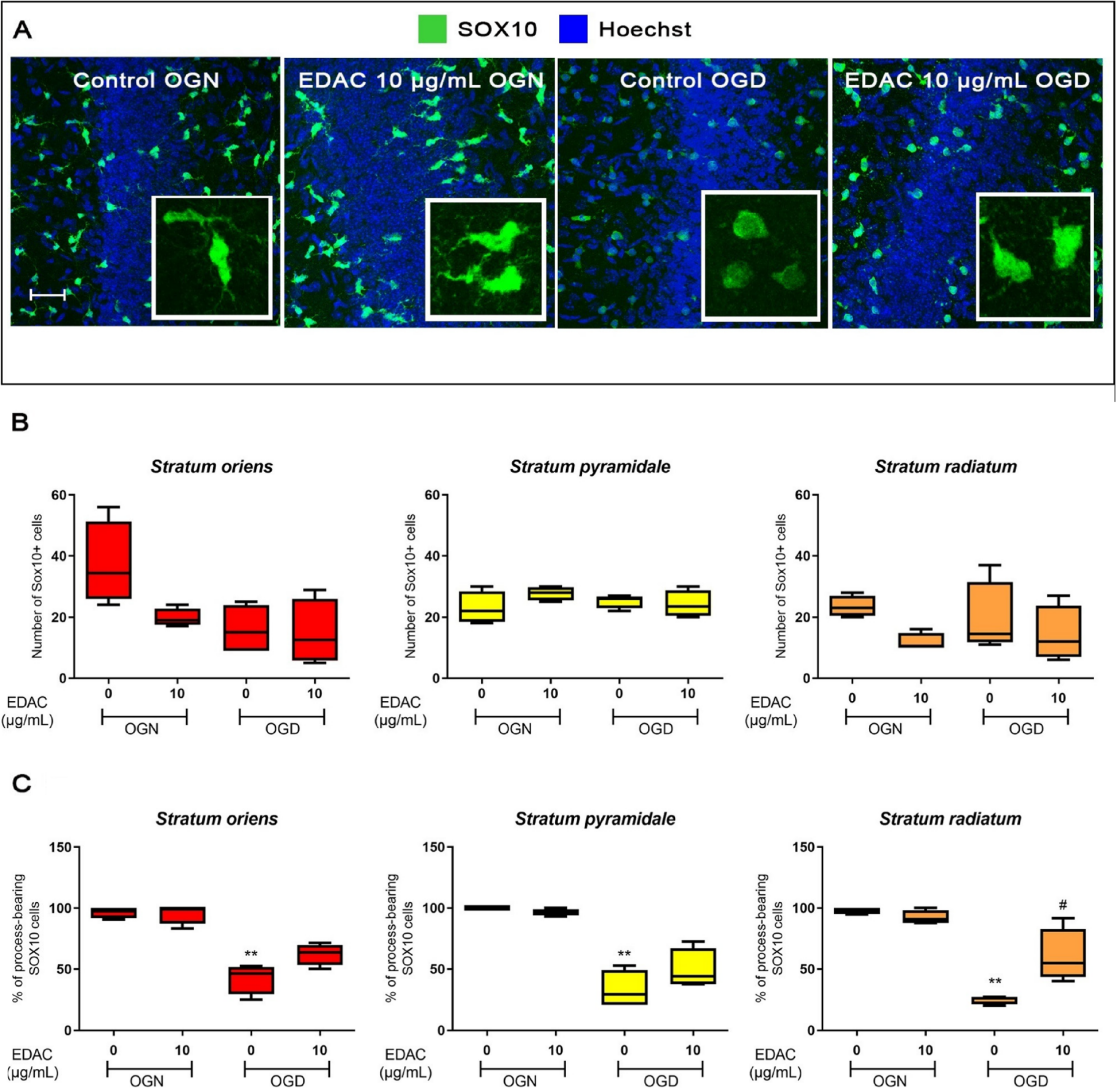
EDAC is cytoprotective for oligodendrocytes in OGD in hippocampal slices. Hippocampal slices from P10-12 SOX10-EGFP mice were incubated in aCSF for 1 h in OGN (normal oxygen + glucose) or OGD (no oxygen or glucose), with EDAC (10 mg/mL), or DMSO vehicle in controls (as indicated). **A** Representative confocal images of the CA1 region of the hippocampus illustrating the effects of OGD and EDAC on SOX10-EGFP + oligodendrocytes (green) counterstained with Hoechst for cell nuclei (blue). Insets are high magnification z-stacks illustrating oligodendrocyte morphology, illustrating that in OGN oligodendrocytes appear normal with bright somata and numerous fine processes, which support myelin sheaths, whereas in OGD there is a marked loss of processes and somata appear dim, and EDAC protected oligodendrocytes against these effects of OGD. Scale bar 50 µm in main panels and 30 μm in insets. **B**, **C** Counts were performed of total SOX10-EGFP + cells (**B**) and process-bearing SOX10-EGFP + cells (**C**, expressed as % of total SOX10-EGFP + cells) in constant FOV in the CA1 strata (as indicated), and plotted as mean ± SEM (*n* = 4 for each treatment group). Data were tested for significance using Kruskal–Wallis test between the control and several samples with post-hoc Dunns Multiple Comparison or standardized with the Mann–Whitney test of variance between two groups; **p < 0.01 indicates comparisons with OGN control, and #*p* < 0.05 indicates comparison with OGD control

Oligodendrocytes are susceptible to ischemia and respond acutely to OGD by the loss of their processes, which is mediated by glutamate excitotoxicity and precedes cellular death [14]. Since EDAC is cytoprotective for astrocytes and stimulates glutamate homeostasis, this led us to examine whether EDAC is also cytoprotective for oligodendrocytes in OGD in hippocampal slices from SOX10-EGFP mice, which enables us to visualise oligodendrocytes and their processes (Fig. 7A) [8]. Hippocampal slices were maintained in OGN or OGD conditions and treated with DMSO vehicle in controls or 10 µg/ mL EDAC, which we show above is the most effective concentration for cytoprotection and stimulating glutamate homeostasis. After 60 min OGN or OGD, slices were analysed for the total number of SOX10-EGFP + oligodendrocytes and counts of oligodendrocytes that were process-bearing compared to those that had lost their processes, which is an early indication of oligodendrocyte damage (Fig. 7A, insets). There was no significant difference in the total number of SOX10-EGFP + cells between the different treatment groups, indicating that oligodendrocytes survive acute OGD and that EDAC is non-toxic for these cells (Fig. 7A, B). In contrast, OGD resulted in a marked loss of processes in oligodendrocytes, with a significant decrease in process-bearing cells in all three strata of the CA1 region, compared to OGN, with 50% less in the *stratum oriens*, and 70% less in the *strata pyramidale* and *radiatum* (Fig. 7A, C; *p* values indicated on the graph). Treatment with EDAC protected oligodendrocytes against this damage in response to OGD (Fig. 7A), and the percentage of process-bearing SOX10-EGFP^+^ cells was not significantly different than OGN controls throughout the CA1 strata (Fig. 7C); EDAC appeared to most effective in *stratum radiatum* where the proportion of process-bearing oligodendrocytes in EDAC (54.9 ± 22%) was significantly greater (*p* < 0.05) than in OGD (26 ± 3.4%) (Fig. 7C). The results indicate that EDAC protect oligodendrocytes from OGD-induced acute cellular damage.

## Discussion

**Fig. 8:**
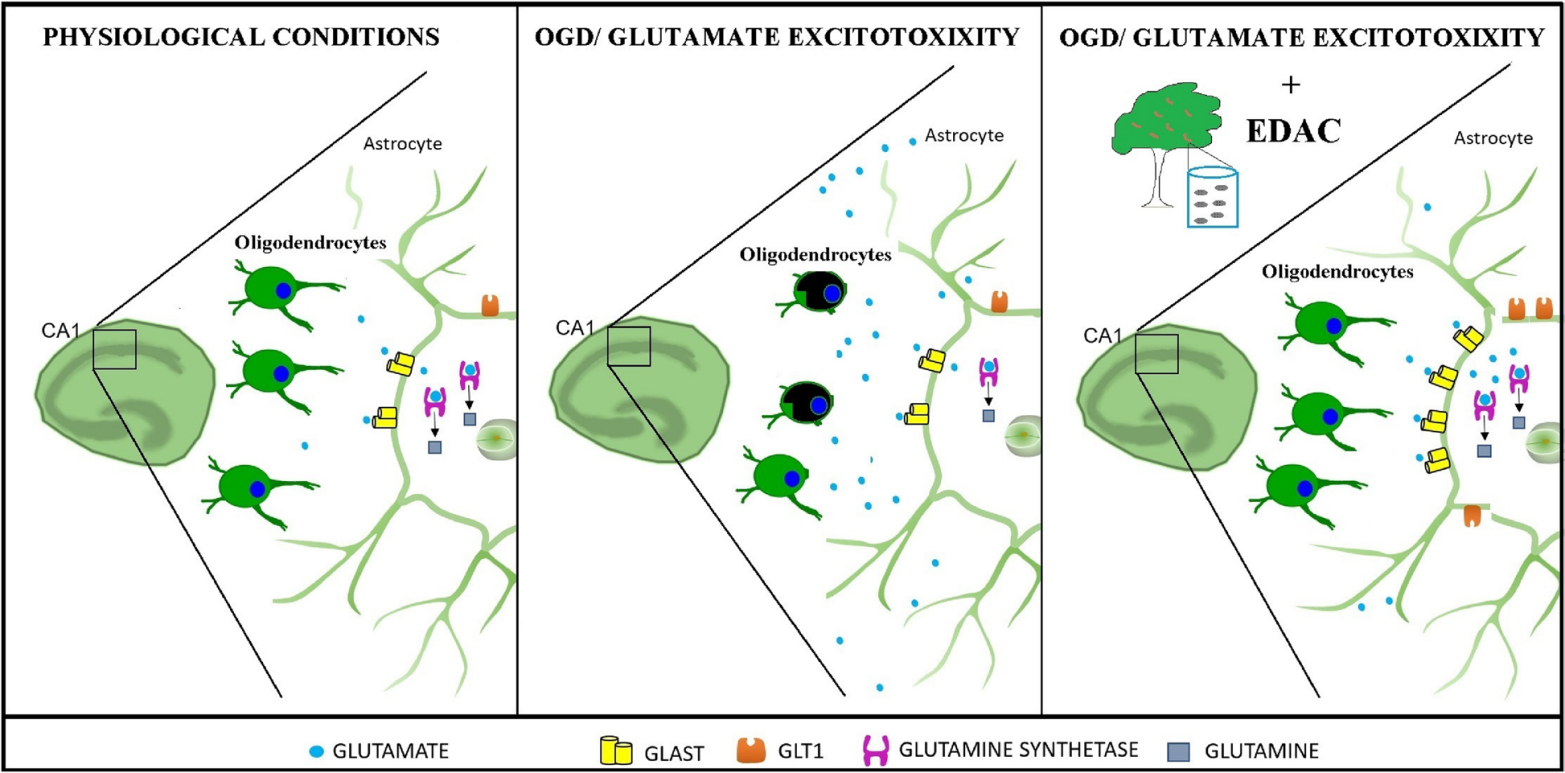
Proposed model of the cytoprotective effects of EDAC. Extract obtained from *Amburana cearensis* seeds (EDAC) is protective against astrocytes and oligodendrocytes in oxygen–glucose deprivation (OGD) and glutamate excitotoxicity in CA1 region of hippocampal slices. The protective effect of EDAC is associated with stimulation of astrocyte increased glutamate homeostasis

A number of constituents in extracts obtained from seeds of *Amburana cearensis* have been shown to be protective against glutamate cytotoxicity in PC12 cells [5] and primary cultures of cerebellar cells [4]. Here, we examined the effect of dichloromethane extract of *A. cearensis* seeds (EDAC) in hippocampal slices. The key finding of this study is that EDAC is cytoprotective against glutamate excitotoxicity and ischemia ex vivo in hippocampus slice cultures by stimulating astrocyte glutamate homeostasis, as summarised in Fig. 8. Glutamate dyshomeostasis is a major factor in stroke and other pathologies, including AD and MS [13], and our results suggest the natural product EDAC could provide a potential adjunct therapy in these diseases.

Our NMR analysis verified that EDAC is rich in coumarin, which encompasses a class of benzopyrones (1,2-benzopyrones or 2H-1-benzopyran-2-ones) phytochemicals and their derivatives. There are over 1,300 subtypes of coumarin and its derivatives and they are known to have antioxidant, anti-inflammatory, anti-bacterial and anti-viral activity [31, 32]. The most recognized pharmacological activity of derivatives from synthetic coumarins is anticoagulant activity, via its VitK inhibitory actions, hence coumarin derivatives are already in use as treatment for stroke and other vascular diseases [33]. Previously, metabolite profiling by Pereira and colleagues [5] using gas chromatography and mass spectrometry found that EDAC is composed of coumarin, methyl esters, γ-sitosterol and ethyl hexadecanoate. Our NMR analysis shows that coumarin is the main constituent of EDAC, comprising approximately one-third of the extract, and the recognised anti-oxidant properties of coumarin are likely to play a key role in the cytoprotective effects of EDAC demonstrated in the present study. Nonetheless, other components of EDAC may also play an important role, and two of them, methyl hexadecanoate and γ-sitosterol, have relevant pharmacological potential in the CNS. For example, methyl hexadecanoate has been shown to improve neuronal survival in the CA1 region of the hippocampus, as well as recovery of learning and memory, in a rat model of global cerebral ischemia [34], and γ-sitosterol was identified in neuroprotective terpenoid-rich extracts from orange juice [35]. Further studies are required to unravel the relative contributions of EDAC constituents to cytoprotection in the CNS, and although it is likely that coumarin is of major importance in the effects observed in the present study, an exciting possibility is that the multiple compounds in EDAC have combinatorial or even synergistic effects.

Due to its maintenance of cell–cell interactions and cytoarchitecture, hippocampal organotypic cultures exposed to glutamate excess have been widely used as an ex vivo model for bioprospection of neuroprotective compounds [21]. Additionally, the CA1 region of the hippocampus receptors is of clinical importance in neuropathology and is highly susceptible to glutamate due to the high density of glutamatergic neurons and NMDA receptors, which are strongly implicated in glutamate-mediated excitotoxicity [36, 37]. Here, we show that EDAC prevents cell death induced by cytotoxic levels of glutamate in hippocampal slices. We also demonstrate that EDAC stimulates astrocyte glutamate homeostatic mechanisms in the face of cytotoxic levels of glutamate. Astrocytes are the main detoxifying cells for excess glutamate, but they are also excited by activation of their NMDA and AMPA receptors [38], which can result in their damage by excess glutamate [39, 40]. In the present study, elevated glutamate induced an overall reduction in GFAP-EGFP in the hippocampus, indicating acute morphological disruption of astrocytes and cell loss, consistent with other studies [17]. Notably, the cytotoxic effect of glutamate on astrocytes was completely overturned by treatment with EDAC, which increased the morphological complexity and overall GFAP-EGFP fluorescence, and an equivalent effect of EDAC was observed in ischemia. Hence, the primary action of EDAC on astrocytes was to protect against cellular atrophy, which is generally associated with downregulation of glutamate transporters and GS and contributes to neuronal and oligodendrocyte demise [13]. GS is pivotal for depleting high levels of glutamate in astrocytes and its upregulation in astrocytes is an important mechanism for neuroprotection against glutamate-mediated excitotoxicity in vitro [20, 21] and in vivo [41]. Here, in addition to a marked increase of GS, we observed increases in glutamate transporters GLT-1 and GLAST in hippocampal organotypic slices treated with EDAC in excitotoxic glutamate. These transporters are expressed by morphologically distinct GFAP astrocytes [42], and their up regulation has been suggested as a neuroprotective mechanism of action for natural compounds against glutamate excitotoxicity [20, 21]. The increased of GFAP, GLT1, GLAST, and GS observed in the slices treated with EDAC + glutamate was associated with an increased number of GFAP-positive cells, which strongly suggests the astrocytes activation as the potential mechanism of EDAC protective effect.

Additionally, the increase in astrocyte processes induced by EDAC reflects greater coverage in the hippocampus and combined with enhanced glutamate homeostasis is a primary mechanism of EDAC cytoprotective activity in high glutamate, applied experimentally or in response to ischemia. Significantly, we show that EDAC also protects oligodendrocytes against ischemia. Oligodendrocyte viability and myelination are disrupted in ischemic stroke [43]. It has previously been shown that OGD induces the loss of oligodendrocyte processes which is mediated by glutamate excitotoxicity and precedes cellular death [14], and similar morphological changes in response to ischemia have been shown in oligodendrocyte precursors [44]. In the present study, we demonstrate OGD induced morphological atrophy in oligodendrocytes in the hippocampus and EDAC significantly protected oligodendroglial cells against these acute responses to ischemia. However, EDAC was not completely cytoprotective for oligodendrocytes, which is consistent with acute ischemic damage to these cells involving multiple mechanisms besides glutamate, including raised extracellular ATP and activation of P2X7 receptors [13, 45].

## Conclusion

In conclusion, EDAC protects the hippocampus from glutamate excitotoxicity and ischemia. An important cytoprotective action of EDAC is to preserve astrocyte and oligodendrocyte integrity by stimulating glutamate homeostatic mechanisms. The results demonstrate EDAC is neuroprotective and support the need for further detailed analyses of the mechanisms underlying its biological effects in complex systems.

## Supplementary Information

**Additional file 1: Supplementary Figure S1.** Chemicalcharacterization of Amburana cearensis extract (EDAC).

**Additional file 2:** **Supplementary Table S1. **Characterization of coumarin chemical structure.

## Data Availability

The authors confirm that the data from which the findings of this study are derived are available within the article.

## Abbreviations

AMPA: α-Amino-3-hydroxy-5-methyl-4-isoxazolepropionic acid
SOX10: SRY-Box Transcription Factor 10
EGFP: Green fluorescent protein
GFAP: Glial fibrillary acidic protein
GS: Glutamine synthetase
GLAST: Glutamate-aspartate transporter
GLT1: Glutamate transporter 1
NMR: Nuclear magnetic resonance
CNS: Central Nervus System
DCM: Dichloromethane
PI: Propidium iodide
ATP: Adenosine triphosphate
NMDA: N-methyl D-aspartate
