# Supplementary figures and images for: Amburana cearensis seed extract stimulates astrocyte glutamate homeostatic mechanisms in hippocampal brain slices and protects oligodendrocytes against ischemia
Source: BMC Complement Med Ther. 2023 May 11;23:154. doi: 10.1186/s12906-023-03959-0 (PMC10173544; doi:10.1186/s12906-023-03959-0)

**Supplementary Figure 1. Chemical characterization of Amburana cearensis extract (EDAC).**


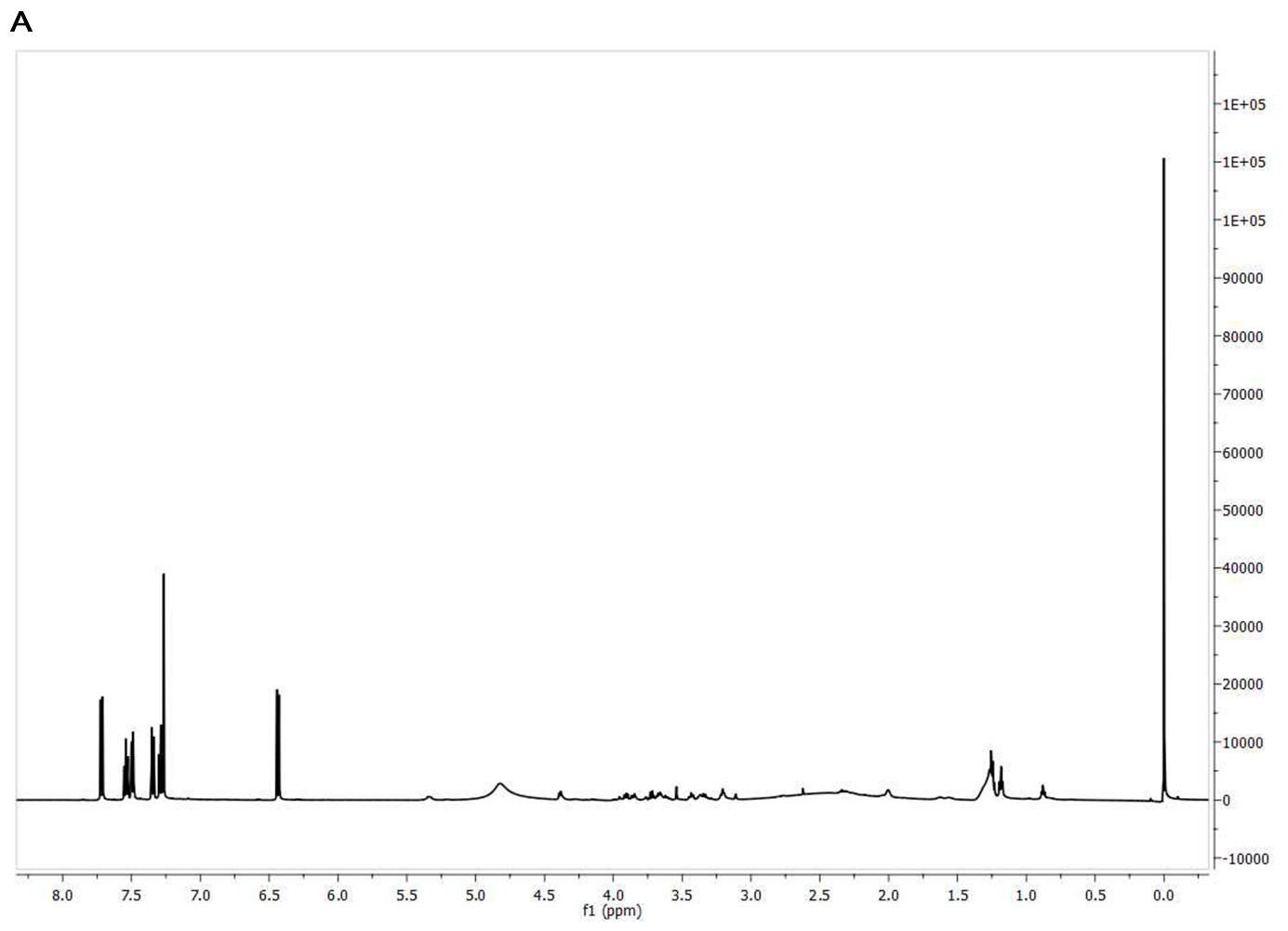


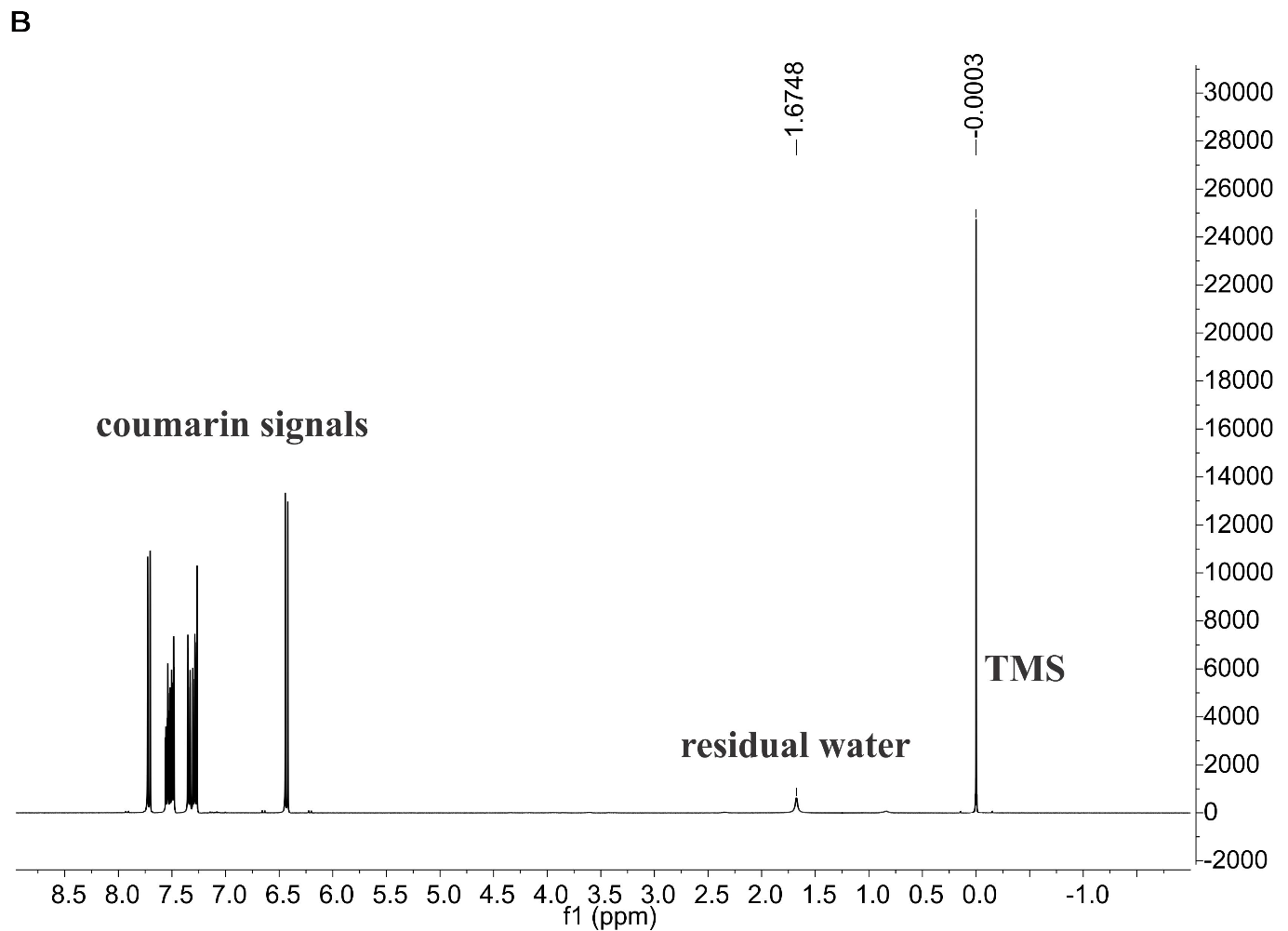


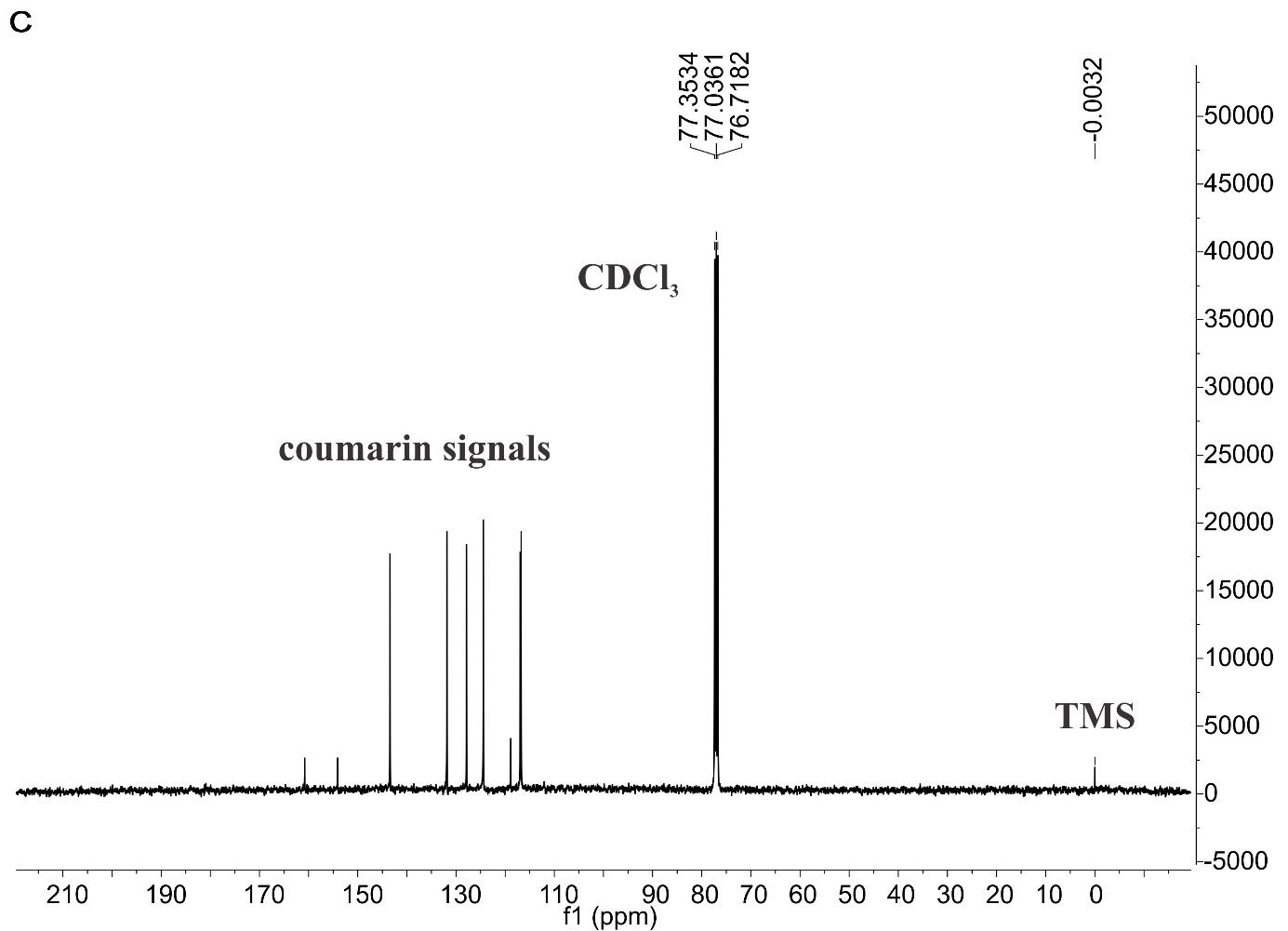


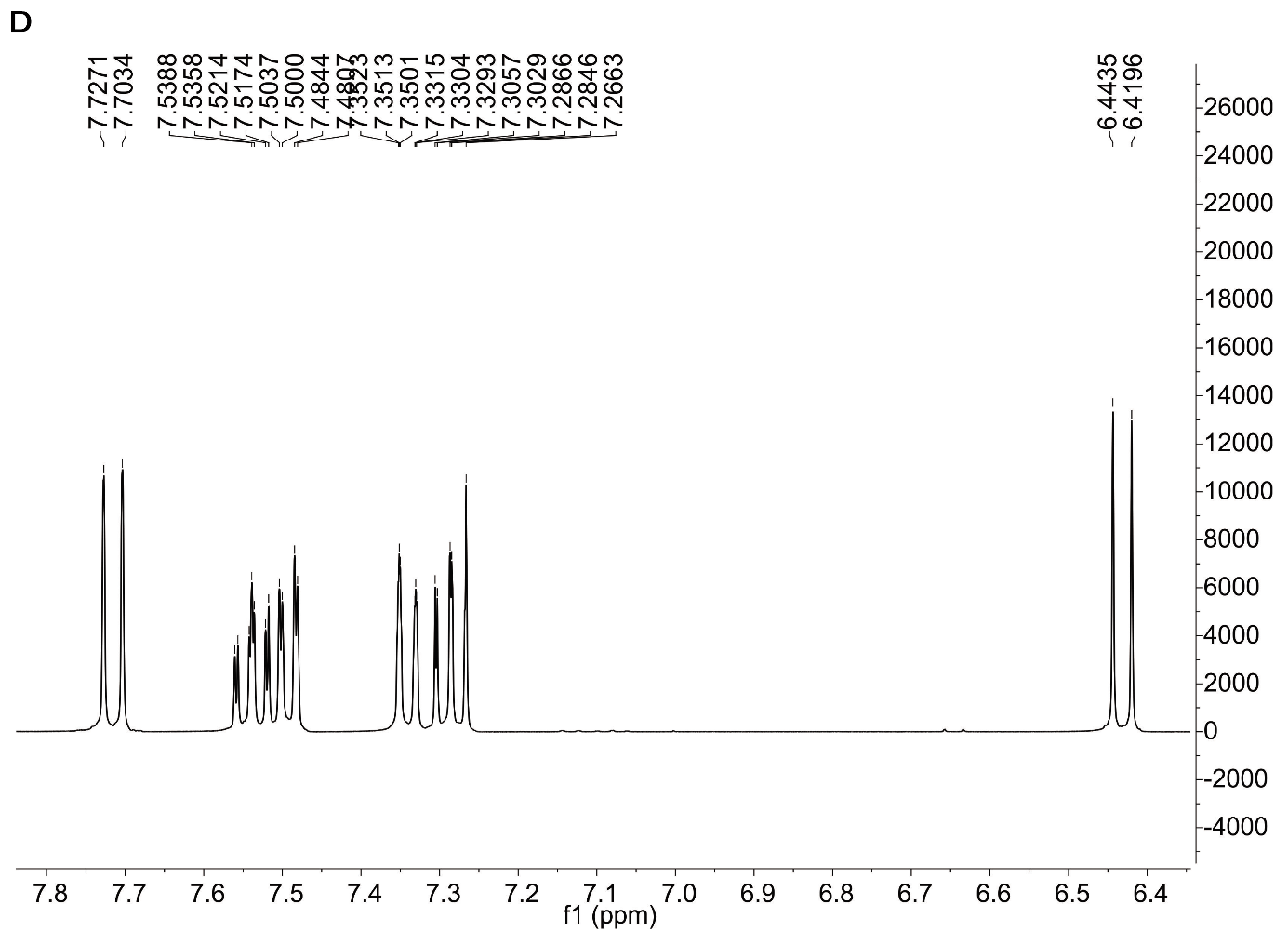


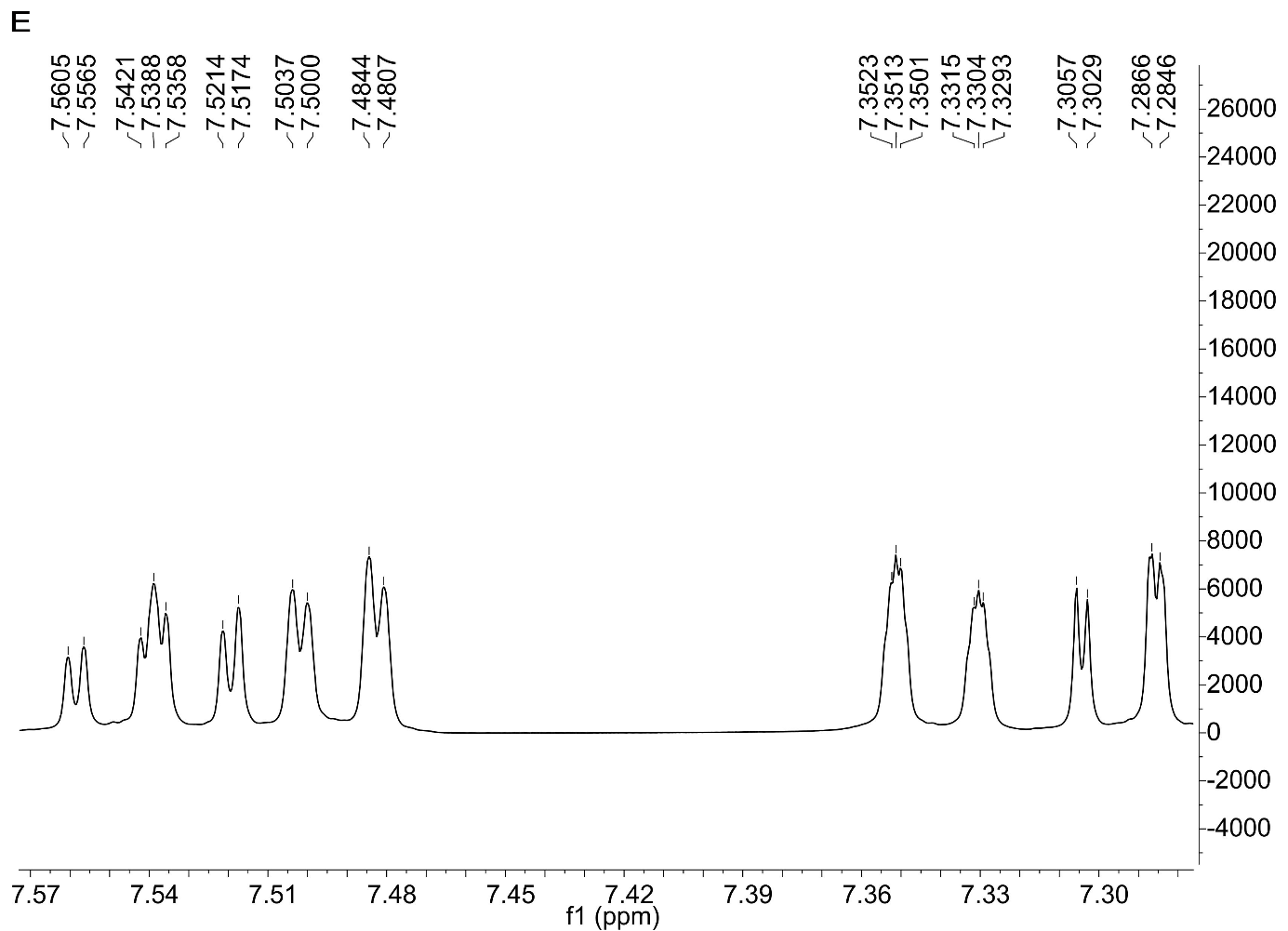


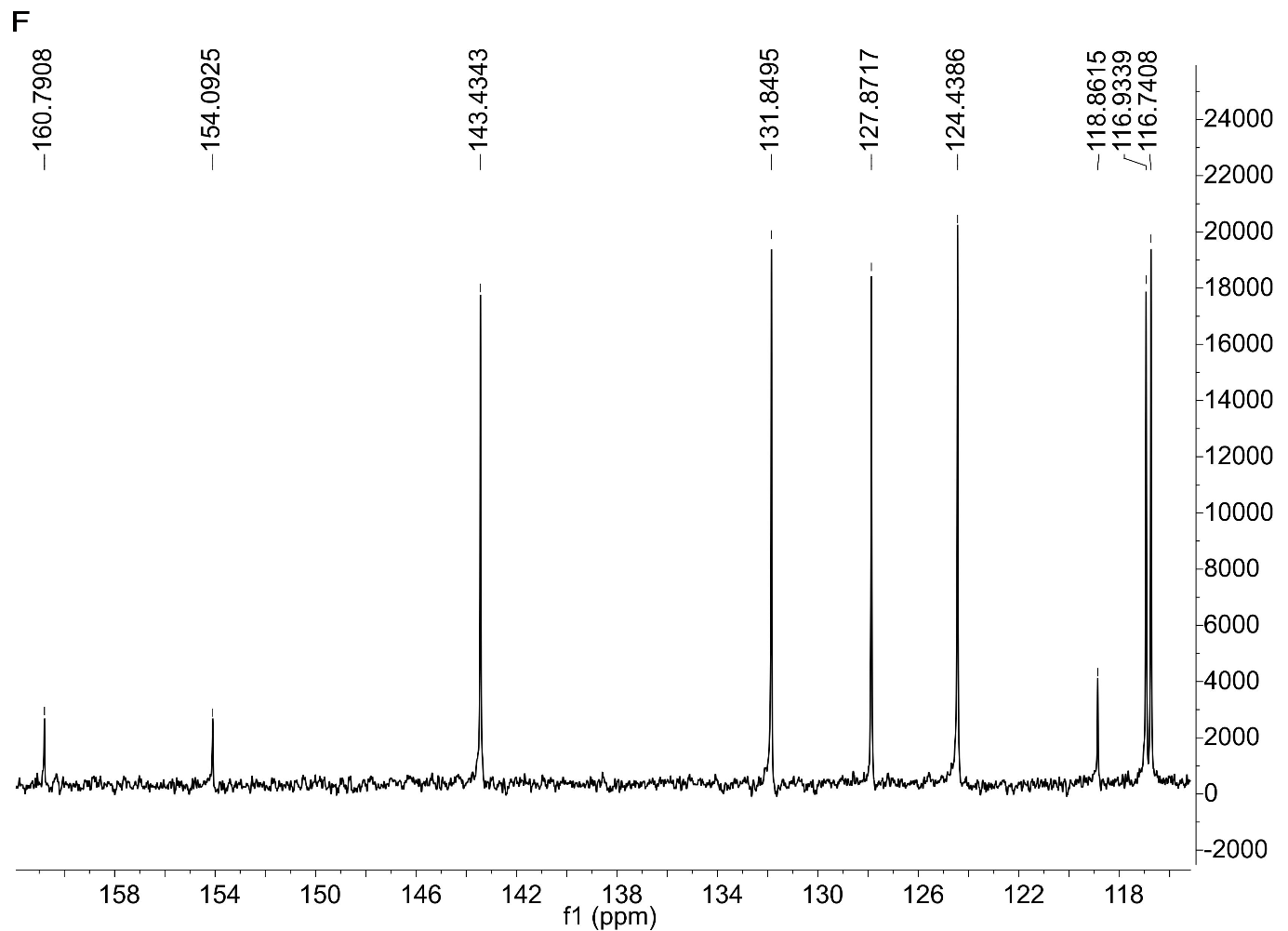

Supplement: Supplementary file 1 — Additional file 1: Supplementary Figure S1. Chemicalcharacterization of Amburana cearensis extract (EDAC). [file 12906_2023_3959_MOESM1_ESM.docx]

**Supplementary Table 1. Characterization of coumarin chemical structure.**


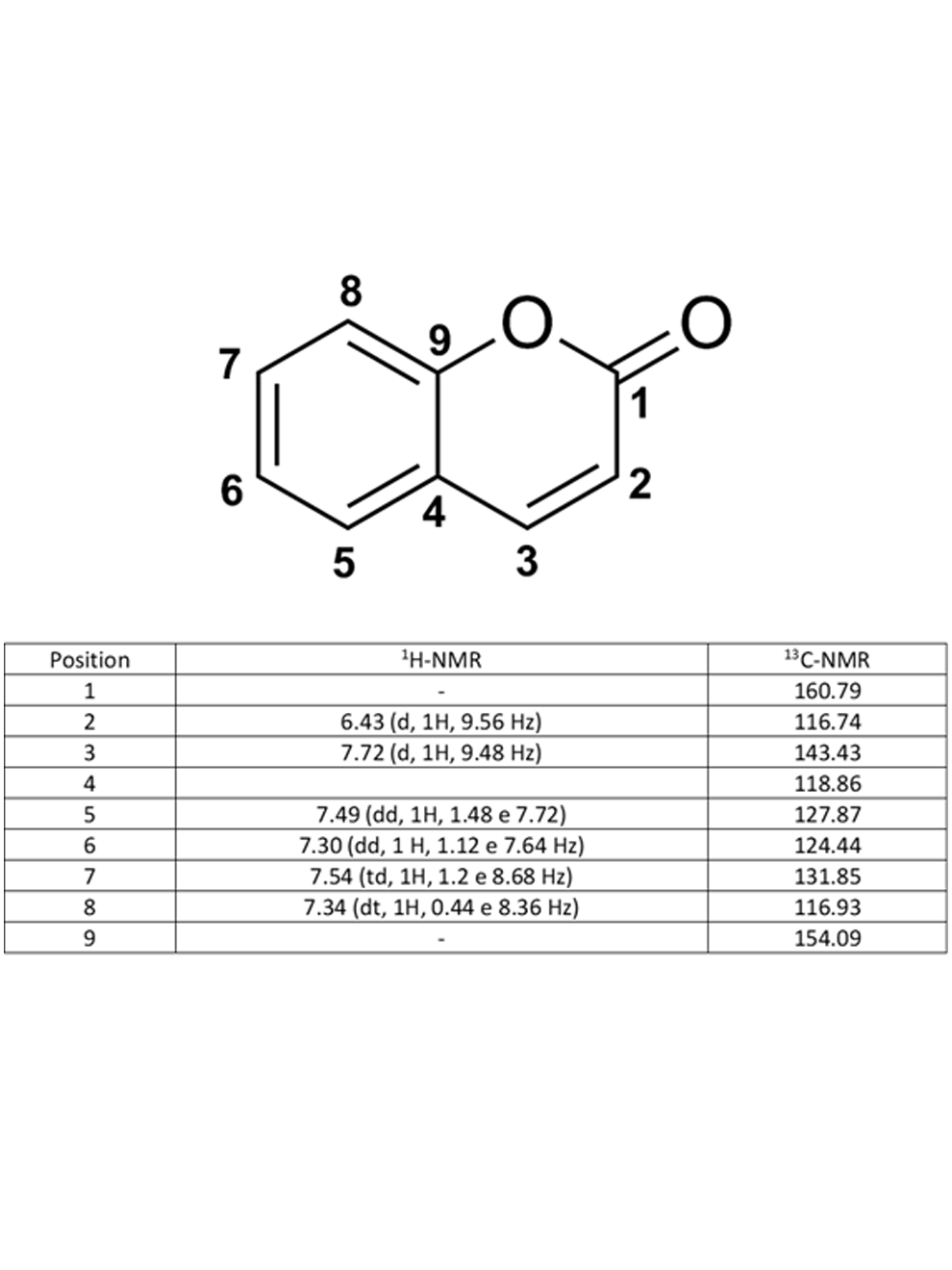

Supplement: Supplementary file 2 — Additional file 2: Supplementary Table S1. Characterization of coumarin chemical structure. [file 12906_2023_3959_MOESM2_ESM.docx]
